# A Linear-Arc Composite Beam Piezoelectric Energy Harvester Modeling and Finite Element Analysis

**DOI:** 10.3390/mi13060848

**Published:** 2022-05-29

**Authors:** Xuhui Zhang, Yan Guo, Fulin Zhu, Xiaoyu Chen, Hao Tian, Hengtao Xu

**Affiliations:** 1 College of Mechanical Engineering, Xi’an University of Science and Technology, Xi’an 710054, China; 20205108039@stu.xust.edu.cn (Y.G.); 20205224060@stu.xust.edu.cn (F.Z.); 19105016004@stu.xust.edu.cn (X.C.); 21205224081@stu.xust.edu.cn (H.T.); 21205016039@stu.xust.edu.cn (H.X.); 2 Shaanxi Key Laboratory of Mine Electromechanical Equipment Intelligent Monitoring, Xi’an University of Science and Technology, Xi’an 710054, China; 3 College of Engineering, Zunyi Normal College, Zunyi 563006, China

**Keywords:** energy harvester, linear-arc beam, dynamic modeling, curvature, finite element method

## Abstract

To improve the output performance of the piezoelectric energy harvester, this paper proposed the design of a linear-arc composite beam piezoelectric energy harvester (PEH-C). First the nonlinear restoring force model of a composite beam was obtained by the numerical simulation method. Afterwards, the corresponding coupled governing equations were derived by using the generalized Hamilton principle, laying the foundation for subsequent in-depth research. After this, a finite element simulation was performed in the COMSOL software to simulate the output voltage, stress distribution, and resonance frequency of the PEH-C under different curvatures. In this way, the effect of curvature change on the PEH-C was analyzed. Finally, the PEH-C with a curvature of 40 $m^{-1}\;$ was prepared, and an experimental platform was built to verify the correctness of the relevant analysis. The results showed that the resonant frequency of the PEH-C can be changed by changing the curvature, and that the stress on the composite beam will increase after the arc segment is introduced. When the curvature of the PEH-C was 40 $m^{-1}$, the open-circuit output voltage was 44.3% higher than that of the straight beam.

## 1. Introduction

With the continuous development of IoT technology, the application scenarios of wireless monitoring nodes are becoming more and more extensive [1,2,3]. It is estimated that by 2025, more than 75 billion IoT-connected infinite monitoring nodes will be put into use [4]. Some of these wireless sensor nodes need to work in harsh environments, such as underground coal mines [5]. A traditional power supply relies on batteries, the practical life of the equipment is limited, and the batteries are difficult to replace [6]. The vibration energy harvesting technology based on the piezoelectric effect has the advantages of a simple structure and high energy density. This technology is expected to solve the problem of power supply for infinite monitoring node equipment [7,8].

The traditional linear cantilever piezoelectric energy harvester has a simple structure and can withstand enormous strain when being subjected to force. Scholars from various countries have carried out extensive research work on this [9,10,11,12]. However, the output power of the linear cantilever piezoelectric energy harvester is relatively low, which significantly limits its practical application. Scholars from all over the world have tried to design structures such as arrays [13] and multiple degrees of freedom [14]. Although these methods improve energy collection performance, the structural volumes are huge and are not conducive to actual use. In addition, some scholars [15,16,17,18,19,20] tried to improve the output efficiency by introducing a nonlinear external force. However, the introduction of an external force coupling still increases the structural complexity and is difficult to apply in some extreme environments. Therefore, it is necessary to optimize the structure of the cantilever beam itself and improve the energy capture efficiency of a single cantilever beam.

Cao [21] designed cantilever beams with two different thickness sections for low-frequency environments. The results show that the output power was 80% higher than traditional straight beams. Wang [22] designed a cantilever beam with varying section thicknesses. A finite element simulation showed that the surface strain of the cantilever beam was larger and more uniform than the traditional straight beam under force. Through experiments, it was verified that the output power of this cantilever beam was increased by 78% compared with the straight beam. Raju [23] designed a piezoelectric energy harvester composed of a rectangular section and a conical section. Experiments showed that the output voltage of this energy harvester was 76.9% higher than that of the traditional straight beam. Muthalif [24] analyzed the influence of the shape and length of the straight beam on the piezoelectric energy harvester through finite element analysis and obtained the optimal solution. Wang [25] determined the relationship between the thickness of the beam and the output power of the energy harvester through theory and experiments. Salem [26] divided the piezoelectric material of the straight beam piezoelectric energy harvester into n segments to widen the frequency and improve the output power.

In addition to optimizing the traditional straight beam structure, Liu [27] developed an L-shaped beam and experimentally proved that it has obvious advantages compared to straight beams. Yang [28] proposed an arched structural beam that is different to traditional straight beams. Simulations show that the arched structure has a larger, uniform stress distribution. Compared with straight beams, arched beams have a higher voltage output and energy conversion efficiency and double-arched beams have the highest energy conversion efficiency. Zhang [29] proposed a linear-arch beam piezoelectric energy harvester. Experiments showed that introducing the arched part can improve the energy harvesting efficiency.

The above studies show that optimizing the cantilever beam structure can improve the output efficiency of piezoelectric energy harvesters. However, no scholars have studied the effect of arc curvature on the output characteristics of cantilever piezoelectric energy harvesters. Therefore, this paper outlines the design of a linear-arc composite beam piezoelectric energy harvester (PEH-C). The corresponding coupled governing equations were derived using the generalized Hamilton principle, laying the foundation for subsequent in-depth research. After this, the finite element simulation was performed in the COMSOL software to simulate the output voltage, stress distribution, and resonance frequency of the PEH-C under different curvatures. The impact of the changes in curvature on the PEH-C were then analyzed. Finally, an experimental platform was set up to verify the correctness of the finite element analysis, which guided the subsequent structure optimization.

## 2. Structure and Theoretical Model of PEH-C

### 2.1. Structure of PEH-C

**Figure 1:**
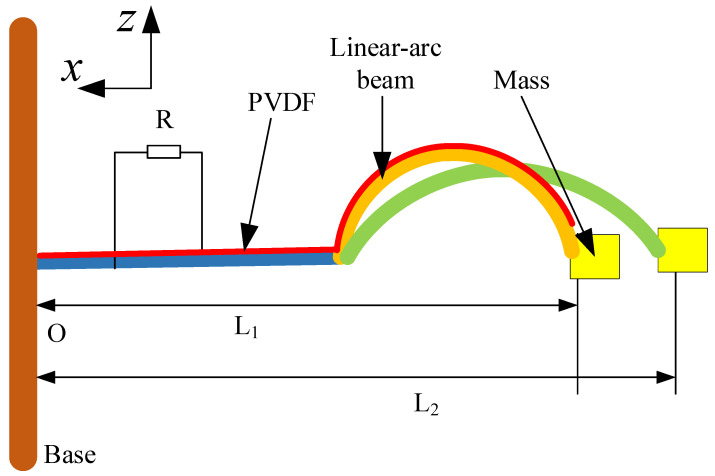
Schematic diagram of PEH-C.

The PEH-C, shown in Figure 1, is comprised of a composite beam, mass, piezoelectric material (PVDF), and base. The cantilever beam is made up of a combination of linear and arced structures. Under the condition of preserving the total length of the structure, the curvature of the curved part can be changed to form a different linear-arc-shaped combination beam. When the curvature is 100 $m^{-1}$, the radius of the arc beam corresponds to 0.01 mm and the corresponding string length is 20 mm. At this point, the curved shape is semi-circular. Earlier team research [30] has proved that this structure has an output power higher than the straight beam. The horizontal distance of the cantilever beam is L. When the curvature of the curved part is changed, the horizontal distance will change accordingly. The mass is fixed at the end of the cantilever beam. The piezoelectric material is attached to the surface of the arc part of the curve-shaped beam to realize energy conversion and the arc part remains free. If the PEH-C is excited by ambient vibrations, the piezoelectric cantilever and mass are vibrated with the base, so the oscillation of the piezoelectric cantilever would result in the deformation of PVDF. Thus, the conversion of mechanical energy from ambiance into electrical energy via the piezoelectric effect can be achieved.

### 2.2. Theoretical Modeling

In order to establish a coupling control equation, this article uses the generalized Hamilton principle.

(1) $$\int_{t_{1}}^{t_{2}}\left[\delta \left(T_{K}-U\right)+\delta W_{nc}\right]dt=0\;$$

where $T_{K}$ is the whole kinetic energy of the proposed system, U is the whole potential energy of the proposed system, and $W_{nc}$ is the external work applied to the system. The whole kinetic energy of the proposed system can be expressed as:

(2) $$T_{K}=\frac{1}{2}\underset{V_{b}}{\int }\rho_{b}{\dot{u}}^{2}\left(X,t\right)dV_{b}+\frac{1}{2}\underset{V_{p}}{\int }\rho_{p}{\dot{u}}^{2}\left(X,t\right)dV_{p}+\frac{1}{2}m_{0}{\dot{u}}^{2}\left(L,t\right)\;$$

where $\;u\left(X,t\right)\;$ is the transverse displacement of the beam; $V_{p}$ and $V_{b}$ are the piezoelectric and substrate layer volume, respectively; $\rho_{p}$ and $\rho_{b}$ are the piezoelectric and substrate layer density, respectively; and $m_{0}$ is the quality of mass.

The whole potential energy of the proposed system can be expressed as:

(3) $$U=W^{*}{}_{b}+W^{*}{}_{p}\;$$

where $W^{*}{}_{b}\;$ is the elastic potential energy of the piezoelectric beam and $W^{*}{}_{p}\;$ is the electric potential energy of the piezoelectric layer. The elastic potential energy of the piezoelectric beam can be expressed as:

(4) $$W^{*}{}_{b}=\frac{1}{2}\underset{V_{b}}{\int }T_{1}S_{1}d_{V_{b}}\;$$

where $T_{1}$ and $S_{1}$ represent the axial stress and the axial strain, respectively. The electric potential energy can be expressed as:

(5) $$W^{*}{}_{p}=\frac{1}{2}\underset{\Omega_{p}}{\int }\left(T_{1}S_{1}-E_{3}D_{3}\right)dV_{p}\;$$

where $E_{3}$ and $D_{3}$ represent the electrical field and the electrical displacement, respectively. The electrical displacement and the axial stress can be expressed as:

(6) $$T_{1}=c_{11}^{E}S_{1}-e_{31}E_{3}\;$$

(7) $$D_{3}=\varepsilon_{33}^{S}E_{3}+e_{31}S_{1}$$

where $\varepsilon_{33}^{S}$ and $e_{31}$ represent the permittivity component at constant strain and the piezoelectric constant, respectively, and $c_{11}^{E}$ is the piezoelectric material elasticity coefficient. The external work applied to the system can be expressed as follows:

(8) $$\delta W_{nc}=-\underset{V_{p}}{\int }f_{i}\delta udV_{p}-Q_{k}\delta v-\underset{V_{b}}{\int }c\dot{u\delta }udV_{b}\;$$

where $f_{i}$ and $Q_{k}$ represent the external incentives and effective current, respectively. In this paper, based on the Rayleigh-Ritz principle, it is assumed that a single-mode approximation of the beam deformation is sufficient and the vibrational displacement of the beam can be expressed as follows:

(9) $$u\left(X,t\right)=\overset{n}{\underset{i=1}{\sum }}\psi_{i}\left(X\right)r_{i}\left(t\right)\;$$

where $\psi_{i}\left(X\right)$ is the $r_{i}\left(t\right)$ mode shape of the beam and $r_{i}\left(t\right)$ is the time-dependent generalized coordinate. Under low-frequency excitations, the vibration of the beam is mainly concentrated in the first-order mode, so it is sufficient to consider one mode to obtain the reduced-order model. Meanwhile, for the boundary conditions where one end is clamped and the other one is free, the allowable function can be written as [31]:

(10) $$\psi_{i}\left(X\right)=1-cos\left[\frac{\left(2i-1\right)\pi x}{2L}\right]\;$$

Substituting Equations (2)–(10) into Equation (1), according to Kirchhoff’s law, the governing equations of the PEH-C system are obtained:

(11) $$M\ddot{r}+C\dot{r}+F_{r}-\theta v=H_{s}\ddot{y}\left(t\right)\;$$

(12) $$\theta \dot{r}+C_{p}\dot{v}+\frac{v}{R}=0$$

where M and C refer to the mass coefficient and the damping coefficient, respectively; θ is the electromechanical coupling coefficient; $C_{p}$ is the capacitance of the piezoelectric patch; and R is the load resistance, as follows:

(13) $$M=\left(\rho_{b}A_{b}+\rho_{p}A_{p}\right)\int_{0}^{L}\psi^{2}\left(X\right)dX+m_{0}\psi^{2}\left(L\right)$$

(14) $$C=cA_{b}\int_{0}^{L}\psi^{2}\left(X\right)dX$$

(15) $$\theta =\frac{ze_{31}A_{p}}{h_{p}}\int_{0}^{L}\psi^{\prime \prime }\left(X\right)dX$$

(16) $$H_{s}=\left(\rho_{b}A_{b}+\rho_{p}A_{p}\right)\int_{0}^{L}\psi \left(X\right)dX+m_{0}\psi \left(L\right)$$

(17) $$C_{p}=\frac{\varepsilon_{33}^{S}b_{p}L_{p}}{h_{p}}$$

where $A_{P}$ and $A_{b}$ represent the piezoelectric and substrate layer cross-sectional area. $F_{r}$ is the nonlinear restoring force of a linear-arc beam. Unlike the linear restoring force of the typical straight beam, the restoring force is nonlinear due to the arced structure in the linear-arced beam. The next step is to use the COMSOL software to numerically calculate the relationship between the force and the displacement of the beam with different curvatures. Finally, curve fitting is carried out to obtain $F_{r}$.

## 3. Finite-Element Simulation

### 3.1. Parameter Settings

Finite element simulation is a widely used and effective numerical analysis method, especially for structural analysis with complex strain and stress. First, 3D modeling and material property settings were performed in COMSOL software. Each parameter is listed in Table 1.

**Figure 2:**
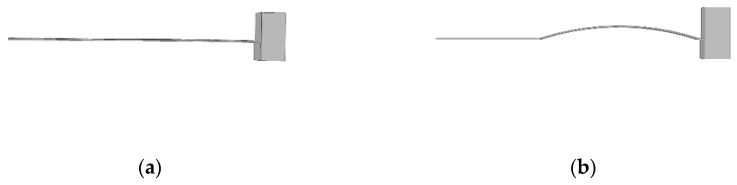

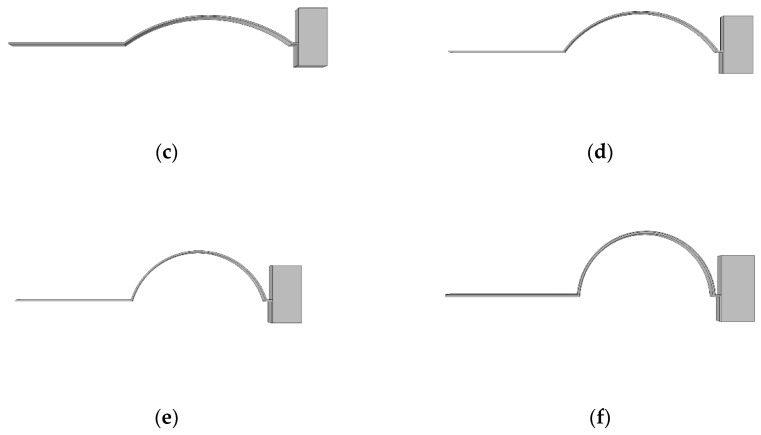
Three-dimensional schematic: (**a**) curvature is 0 $m^{-1}$; (**b**) curvature is 20 $m^{-1}$; (**c**) curvature is 40 $m^{-1}$; (**d**) curvature is 60 $m^{-1}$; (**e**) curvature is 80 $m^{-1}$; (**f**) curvature is 100 $m^{-1}$.

The whole frame is schematically shown in Figure 2. Figure 2a to Figure 2f show the schematic diagrams of curvature from 0 $m^{-1}$ to 100 $m^{-1}$, respectively.

**Figure 3:**
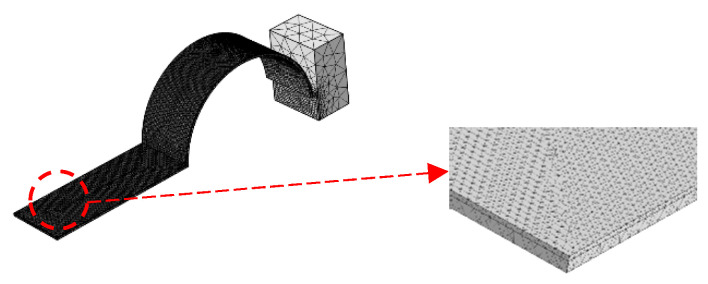
Schematic diagram of mesh division.

After meshing, the type of module was a free tetrahedral. Because the PVDF was thin, in order to ensure the accuracy of numerical simulation, the PVDF was subjected to grid refinement processing. The end mass was less relevant to the finite element analysis in this paper, so the mesh division was sparse and the final division result is shown in Figure 3.

**Figure 4:**
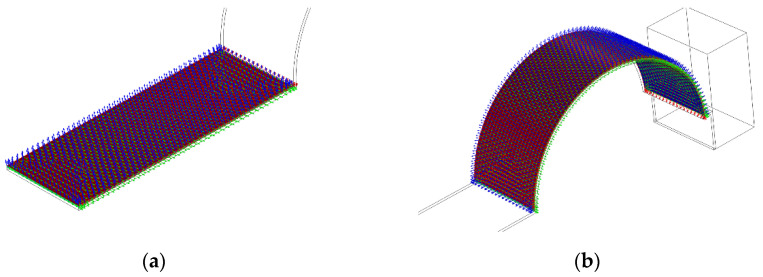
Schematic diagram of material coordinate system: (**a**) straight beam part; (**b**) arc beam section.

During the finite element analysis, the PVDF of the straight beam part and the arc part have different polarization directions. The working mode of the piezoelectric material PVDF in the piezoelectric energy harvester was the D31 mode, so the polarization direction of the PVDF in the arc part should have been polarized along the radial direction. Therefore, the two material coordinate systems needed to be set separately and these coordinate systems are shown in Figure 4. For the convenience of calculation, the material coordinate system was simplified to assume orthogonality.

### 3.2. Finite Element Analysis Mesh Accuracy Verification

**Figure 5:**
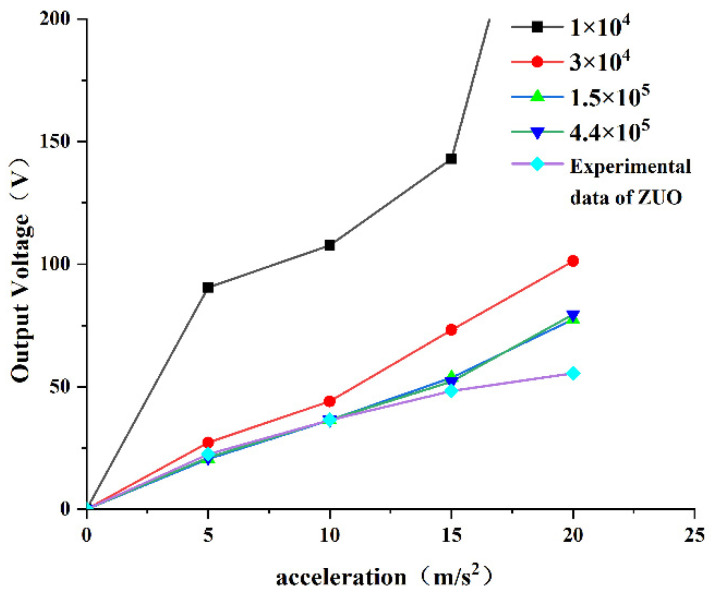
Voltage-acceleration diagram.

In order to verify the validity of the finite element simulation in this paper and the accuracy of the meshing accuracy, the PEH-C with a curvature of 100 $m^{-1}$ was simulated first. In this paper, four different numbers of grids were selected for calculation and the simulated resonant frequency and open-circuit output voltage were compared with the experimental results of Zuo [32]. Figure 5 shows the relationship between excitation and open-circuit output voltage for different mesh divisions. The results showed that under low acceleration excitation, when the number of grids increased from 1 × 10^4^ to 4.4 × 10^5^, the output voltage in the figure kept approaching the experimental results of Zuo. When the number of grids was 1 × 10^4^, the mesh height was larger than the PVDF height, the simulation error was too large. When the number of grids was increased from 1.7 × 10^5^ to 4.4 × 10^5^, the results were not much different. Therefore, we chose the number of meshing elements as 4.4 × 10^5^ for follow-up research. However, as the acceleration continued to increase, the simulation results deviated from the experimental results, which may have been due to the obvious nonlinear characteristics of the beam under high excitation in the experiment.

In summary, in order to ensure the accuracy of the finite element simulation, the number of meshes in the subsequent finite element numerical calculation was set to 1.7 × 10^5^ and the given acceleration excitation was set to 5 $m/s^{2}$.

### 3.3. Nonlinear Restoring Force of the Linear-Arc Beam

**Figure 6:**
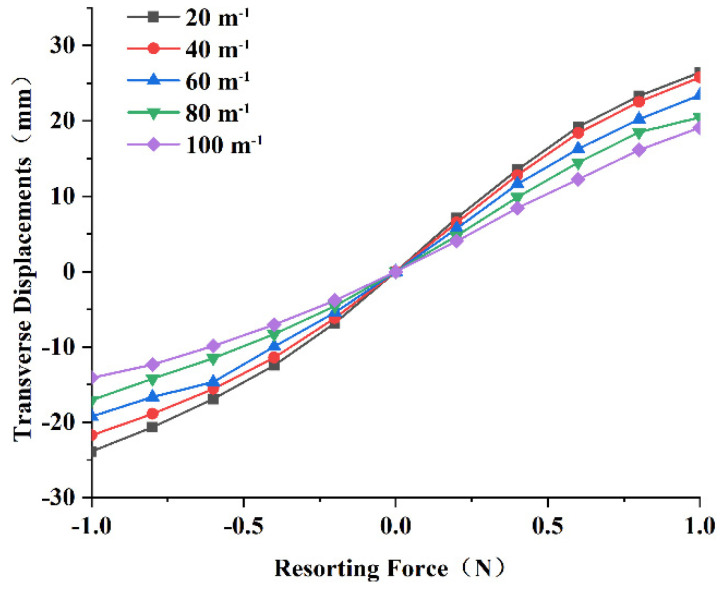
Displacement-restoring force curve of the curve-shaped beam.

In order to obtain the nonlinear restoring force of a linear-arc beam, we used the COMSOL software to perform a steady-state study, apply a force in the z-direction on the mass, and obtain the displacement-restoring force curve of the curve-shaped beam. The results are shown in Figure 6. The relationship between the restoring force and transverse displacements was then fit to a polynomial, as follows:

(18) $$F_{r}=k_{1}u^{3}\left(L,t\right)+k_{2}u^{2}\left(L,t\right)+k_{3}u\left(L,t\right)$$

where $k_{1}$, $k_{2}$, and $k_{3}$ are constant coefficients on the third-, second-, and first-order terms, respectively. 

The result shows that when the curvature is 20 $m^{-1}$, the nonlinear restoring force of the linear-arc beam can be expressed as:

(19) $$F_{r}=27.23u^{3}\left(L,t\right)-0.15u^{2}\left(L,t\right)+0.02u\left(L,t\right)$$

The result shows that when the curvature is 40 $m^{-1}$, the nonlinear restoring force of the linear-arc beam can be expressed as:

(20) $$F_{r}=33.26u^{3}\left(L,t\right)-0.35u^{2}\left(L,t\right)+0.04u\left(L,t\right)$$

Substituting Equation (19) or (20) into Equation (11), the governing equations of the PEH-C system with curvatures of 20 $m^{-1}\;$ and 40 $m^{-1}$ were obtained, respectively. The equations for other curvatures could also be deduced by analogy. The corresponding coupled governing equations were derived by using the generalized Hamilton principle, laying the foundation for subsequent in-depth research.

### 3.4. Finite Element Analysis

#### 3.4.1. Resonant Frequency

**Figure 7:**
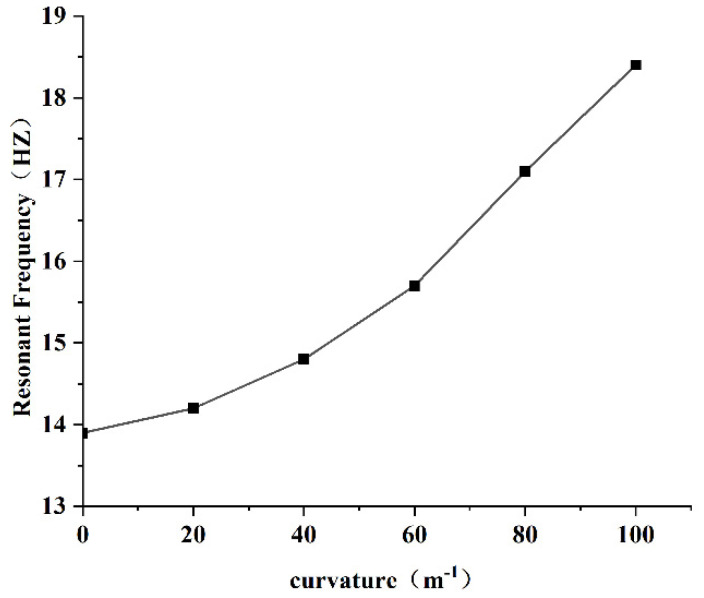
Schematic diagram of resonant frequency.

In order to obtain the relationship between curvature and resonant frequency, the PEH-C with curvatures of 0 $m^{-1}$, 20 $m^{-1}$, 40 $m^{-1}$, 60 $m^{-1}$, 80 $m^{-1}$, and 100 $m^{-1}$ were selected for research. The results are shown in Figure 7.

It is evident from Figure 7 that the resonant frequency of the PEH-C gradually increased with the increase in curvature. It can be seen that under the condition of keeping the length unchanged, the curvature of the curved beam part increased, the bending radius decreased, and the corresponding stiffness increased, resulting in an increase in the resonance frequency. Conversely, when the curvature was smaller, the curved beam was closer to the straight beam, the stiffness decreased, and the resonance frequency decreased accordingly.

#### 3.4.2. Stress Distribution

**Figure 8:**
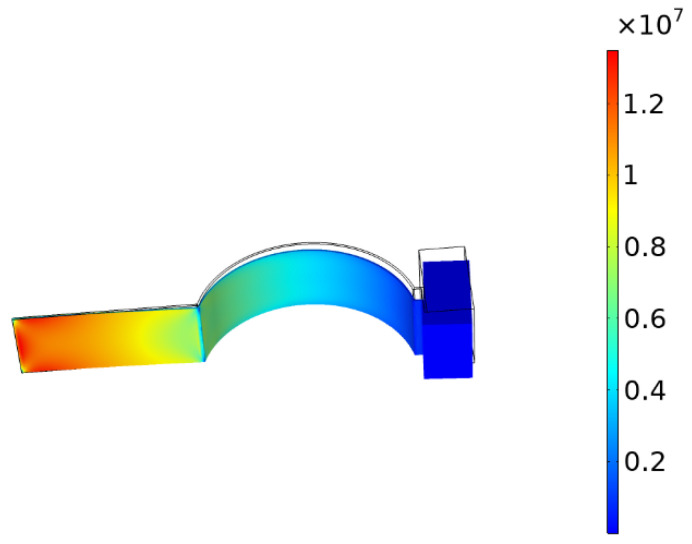
Stress cloud.

In order to explore the effect of curvature on the stress distribution, the curvature was unchanged. The acceleration magnitude was set to 5 $m/s^{2}$ and the direction was taken along the Z-axis. Taking the curvature of 100 $m^{-1}$ as an example, the stress cloud diagram is shown in Figure 8.

**Figure 9:**
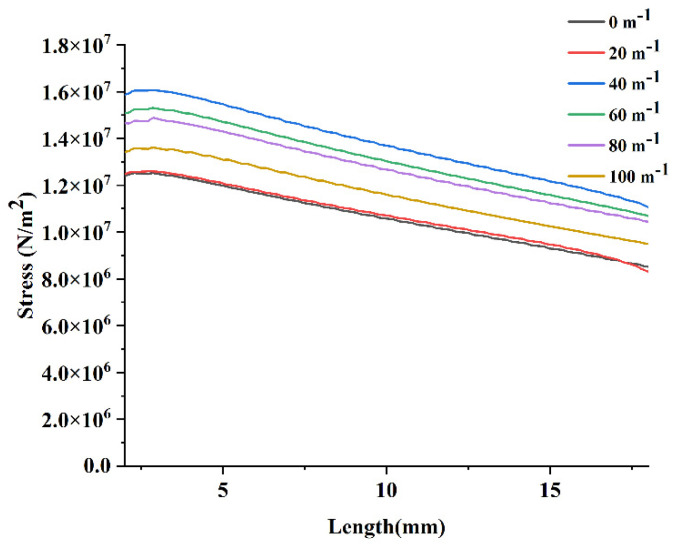
Stress-length diagram of the straight segment.

The stress on the midline of the PEH-C straight beam was represented as a stress–length diagram. As a result, as shown in Figure 9, the stress gradually decreased from the fixed end to the free end of the beam. There was no significant difference between the PEH-C with a curvature of 20 $m^{-1}$ and a straight beam with a curvature of 0 $m^{-1}$ in the straight section. When the curvature of the PEH-C was increased from 20 $m^{-1}$ to 40 $m^{-1}$, the stress under the same load increased significantly. After this, as the curvature increased from 40 $m^{-1}$ to 100 $m^{-1}$, the stress decreased. The stress reached its peak when the curvature was 40${\;m}^{-1}$.

**Figure 10:**
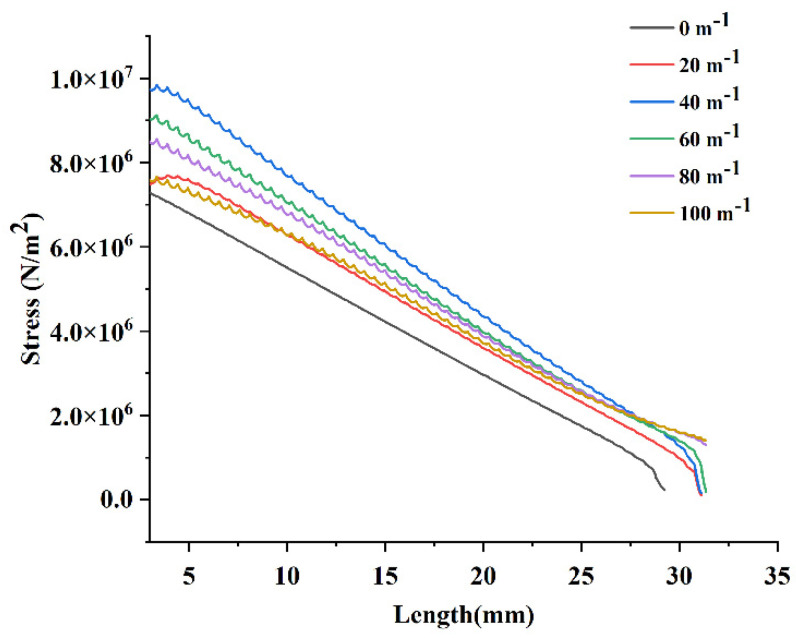
Stress-length diagram of the arc segment.

The stress on the midline of the PEH-C arc beam was also represented as a stress–length diagram. As a result, as shown in Figure 10, the stress gradually decreased from the fixed end to the free end of the beam. The application of the stress law of the PEH-C in the arc segment was the same as that in the straight segment. The stress was largest at 40 $m^{-1}$. The stress then decreased uniformly when the curvature changed from 60 $m^{-1}$ to 100 $m^{-1}$. The stress distribution with a curvature of 100 $m^{-1}$ was slightly uniform, but the average stress was small. Through finite element analysis, it was observed that, compared with straight beams, the PEH-C has obvious advantages in stress distribution. A straight beam with a curvature of 0 $m^{-1}$ and a PEH-C with a curvature of 20 $m^{-1}$ had similar stress distributions in the straight section, but the stress in the arc section was significantly different. The stress on straight beams decreased linearly and the stress on the PEH-C with a curvature of 20 $m^{-1}$ was significantly higher than that on straight beams.

#### 3.4.3. The Output Voltage

**Figure 11:**
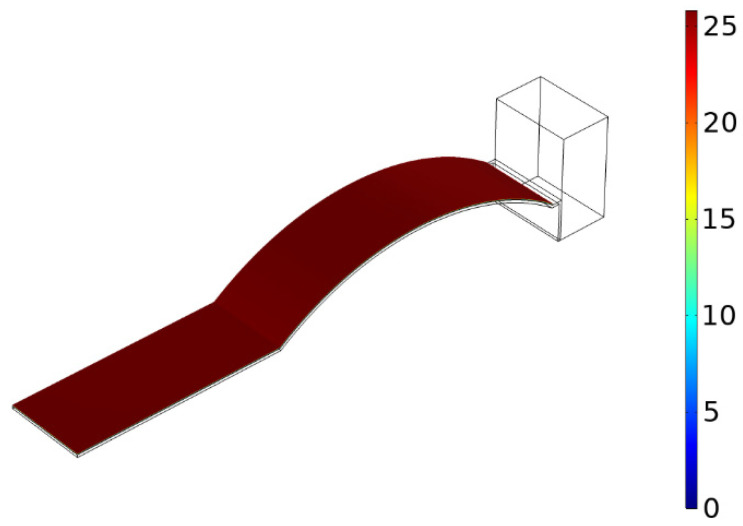
Voltage cloud.

In order to explore the effect of the curvature on the output voltage, the curvature was unchanged. The acceleration magnitude was set to 5 $m/s^{2}$ and the direction was along the Z-axis. The voltage cloud diagram is shown in Figure 11.

**Figure 12:**
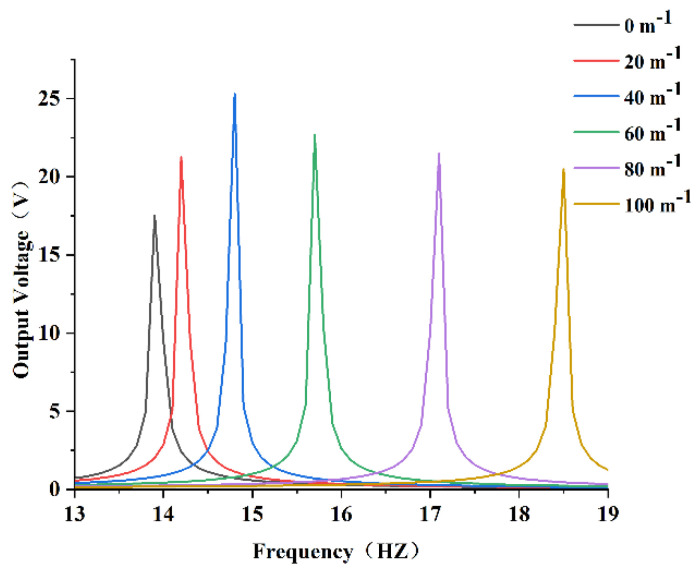
Output voltage.

The voltage output results of the PEH-C with different curvatures are shown in Figure 12. With the increase in curvature, the maximum output voltage showed a trend of increasing first and then decreasing, reaching a peak value when the curvature was 40 $m^{-1}$ and the voltage was about 25 V. Compared with the PEH-C with a curvature of 100 $m^{-1}$, the output voltage increased by 23.6%. Compared with the straight beam, the output voltage increased by 44.3%.

## 4. Experimental Validation

**Figure 13:**
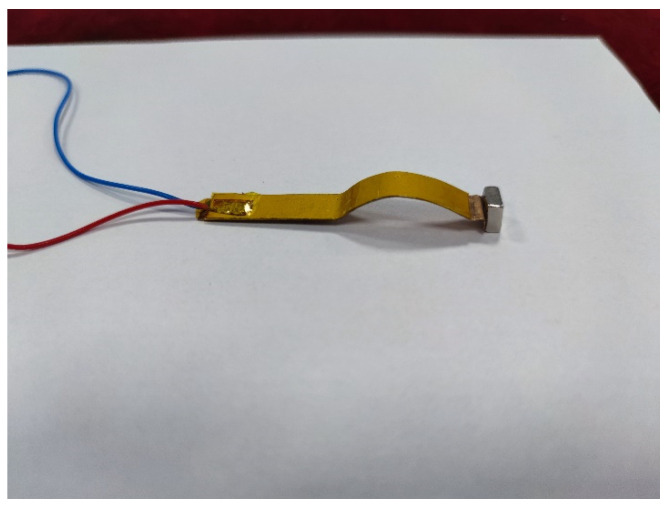
PEH-C with 40 $m^{-1}$ curvature.

**Figure 14:**
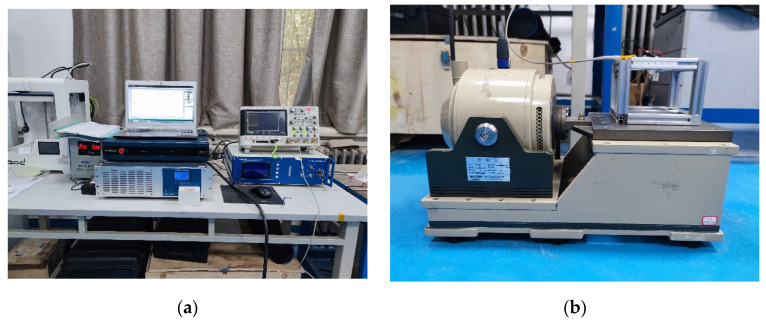
Experimental platform: (**a**) control section; (**b**) vibration table with PEH-C.

In order to verify the correctness of the above theory, first, the optimal solution of the curvature of finite element analysis was selected and the beam with a curvature of 40 $m^{-1}$ was prepared. The width of the prepared composite beam was 8 mm, the height was 0.2 mm, the length of the straight section was 20 mm, the radius and chord length of the arc part were 25 mm and 29.4 mm, respectively, and the arc height was 9.5 mm. The width of the PVDF pasted on the composite beam was 8 mm, the thickness was 0.11 mm, the volume width of the end mass was 8 mm, the height was 8 mm, and the length was 5 mm, as shown in Figure 13. Then, the experimental platform was built. In the experiment, the excitation signal was set by the computer and the sinusoidal signal was sent out by the vibration controller (VT-9008), which was amplified by the power amplifier (GF-20) and output to the vibration table (E-JZK-5T). The vibration table operated according to the preset excitation signal. The experimental device is shown in Figure 14.

**Figure 15:**
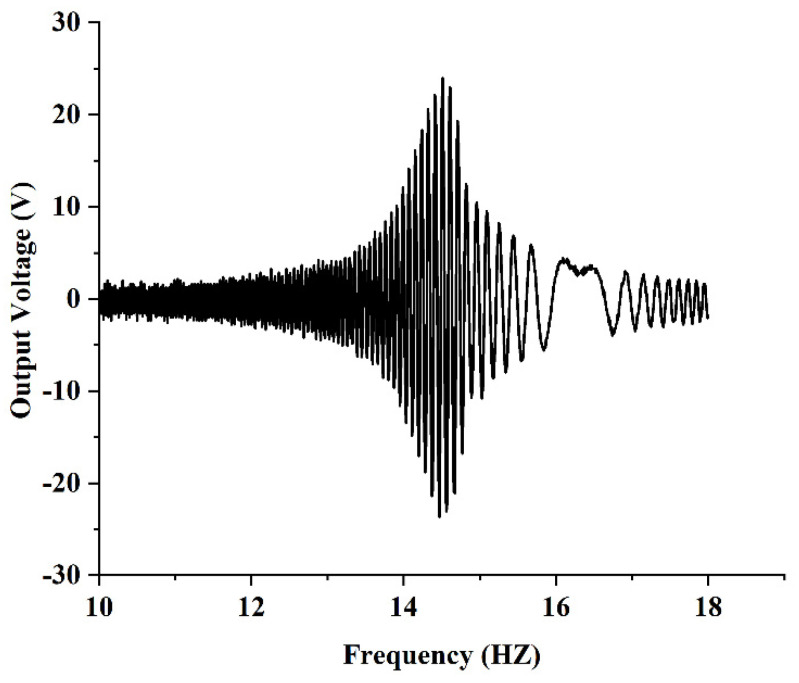
Frequency sweep experiment.

**Figure 16:**
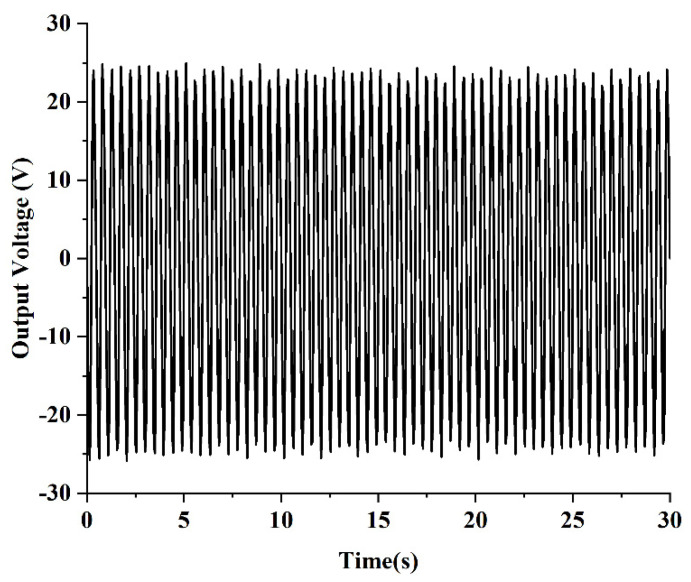
Residency experiment.

Under the given excitation conditions, the output end of the PEH-C was connected directly to the oscilloscope probe (open circuit) and the required output voltage signal was obtained through the oscilloscope. First, the excitation amplitude of the exciter table was set to 5 $m/s^{2}$ and the excitation frequency to 10–18 Hz and a frequency sweep experiment was carried out under simple harmonic excitation. Figure 15 shows the frequency–voltage diagram collected by the oscilloscope. It can be seen from the diagram that when the frequency was 14.5 HZ, the output voltage reached 24 V. After calculation, the resonant frequency measured in the experiment was 3% different from the simulation result. The maximum voltage was 4.2% different from the simulation result. After that, the excitation amplitude of the exciter table was set to 5 $m/s^{2}$ and the excitation frequency to 14.5 Hz and a dwell experiment was carried out under the condition of the PEH-C resonance. The experimental results are shown in Figure 16. At a frequency of 14.5 HZ, the open-circuit output voltage amplitude was stable at about 24 V. Compared with the PEH-C device, with a curvature of 100 $m^{-1}$, the open-circuit output voltage amplitude of the PEH-C with a curvature of 40 $m^{-1}$ was increased by 9%.

## 5. Conclusions

This paper outlines a design for a linear-arc composite beam piezoelectric energy harvester (PEH-C). The corresponding coupled governing equations were derived using the generalized Hamilton principle, laying the foundation for subsequent in-depth research. After this, the finite element simulation was performed in the COMSOL software to simulate the output voltage, stress distribution, and resonance frequency of the PEH-C under different curvatures. The impact of changes in the curvature on the PEH-C were then analyzed. Finally, the PEH-C with a curvature of 40 $m^{-1}$ was prepared and an experimental platform was built to verify the correctness of the relevant analysis. The following main conclusions were obtained from the simulation and experiment:

(1) The PEH-C was numerically simulated using COMSOL software to determine the relationship between the nonlinear restoring force and the transverse displacements. Then, curve fitting was performed to obtain the equation for the nonlinear restoring force. Finally, the corresponding coupled governing equations were derived by using the generalized Hamilton principle.
(2) The resonance frequency of the PEH-C gradually increased with an increase in curvature. It was observed that, under the condition of keeping the length unchanged, the curvature of the curved beam part increased, the bending radius decreased, and the corresponding stiffness increased, resulting in an increase in the resonance frequency. Conversely, when the curvature was smaller, the curved beam was closer to the straight beam, the stiffness decreased, and the resonance frequency decreased accordingly.
(3) Compared with the straight beam, the PEH-C introduced into the arc segment was subjected to greater stress under the same excitation. Under the same excitation, the PEH-C with a curvature of 40 $m^{-1}$ had the largest stress and the PEH-C with a curvature of 100 $m^{-1}$ had relatively uniform stress.
(4) The finite element simulation results showed that the PEH-C with a curvature of 40 $m^{-1}$ had the best open-circuit voltage output performance. Experiments showed that the open-circuit voltage output performance of the PEH-C with a curvature of 40 $m^{-1}$ was 9% higher than that of the PEH-C with a curvature of 100 $m^{-1}$.

## Figures and Tables

**Table 1:** Material parameters for simulation.

Parameter	Value	Unit
Mass density	7800	$\mathrm{kg}/m^{3}$
Mass size	8 × 8 × 5	mm
PVDF density	1780	$\mathrm{kg}/m^{3}$
PVDF Piezoelectric stress	11.5	$C/m^{2}$
PVDF elastic modulus	3	GPa
PVDF height	0.11	mm
PVDF overall length	51.4	mm
PVDF width	8	mm
Substrate layer density	8300	$\mathrm{kg}/m^{3}$
Substrate layer elastic modulus	128	GPa
Substrate layer height	0.2	mm
Substrate layer overall length	51.4	mm
Substrate layer width	8	mm

## Data Availability

Not applicable.
